# Future Directions for Public Health Education Reforms in India

**DOI:** 10.3389/fpubh.2014.00068

**Published:** 2014-09-23

**Authors:** Sanjay P. Zodpey, Himanshu Negandhi, Rajiv Yeravdekar

**Affiliations:** ^1^Public Health Foundation of India, New Delhi, India; ^2^Faculty of Health and Biomedical Sciences, Symbiosis International University, Pune, India

**Keywords:** public health education in India, public health education reforms, future of public health education, public health professionals, education of health professionals

## Abstract

Health systems globally are experiencing a shortage of competent public health professionals. Public health education across developing countries is stretched by capacity generation and maintaining an adequate ‘standard’ and ‘quality’ of their graduate product. We analyzed the Indian public health education scenario using the institutional and instructional reforms framework advanced by the Lancet Commission report on Education of Health Professionals. The emergence of a new century necessitates a re-visit on the institutional and instructional challenges surrounding public health education. Currently, there is neither an accreditation council nor a formal structure or system of collaboration between academic stakeholders. Health systems have little say in health professional training with limited dialogue between health systems and public health education institutions. Despite a recognized shortfall of public health professionals, there are limited job opportunities for public health graduates within the health system and absence of a structured career pathway for them. Public health institutions need to evolve strategies to prevent faculty attrition. A structured development program in teaching–learning methods and pedagogy is the need of the hour.

## Need for Reforms in Health Professional Education

The role of health systems is extremely vital in the global movement toward Universal Health Coverage (UHC). To achieve UHC, an inclusive health-care system reaching every community, including the poorest and hardest to access (1), is essential. Health systems globally face a variety of challenges, but they are especially stretched along their most important structure: human capital. Human resources for health are an important building block of health systems as recognized by the WHO (2). In spite of this recognition and seven years of time, over a billion people worldwide continue to lack access to quality health services – in large part because of a huge shortage, imbalanced skill mix, and uneven geographical distribution of health workers (3). WHO estimated a shortfall of nearly 7.2 million health workers worldwide, which is expected to rise to 12.9 million by 2035 (4).

Public health education is a fundamental tool to create public health professionals. The supply side of the health systems struggles to create an ever-increasing number of public health professionals, while at the same time maintaining an adequate “standard” and “quality” of their graduate product. While the former can be linked to increasing the planned expenditures into expanding educational programs so as to produce a higher number of graduates, the latter needs a complete change in the outlook of educational systems and processes. The Lancet Commission Report, “*Health professionals for a new century: transforming education to strengthen health systems in an interdependent world*,” brought together professional and academic leaders to develop a shared vision and common strategy for postsecondary education in medicine, nursing, and public health at the global level and free from the silos of individual professions (5). This sets across a global reforms agenda that would result in a transformative learning experience rather than formative and informative learning alone and an interdependence in education.

In India, gaps prevail in understanding the overall dynamics of public health education, both its quantity and quality. Like the general health workforce, the challenges that surround the public health workforce are centered around a numerical deficiency (6); competencies and expectations poorly suited to the health needs of the populations they serve (3); and a skill-mix imbalance against the background of negative work environments and a weak knowledge base (7).We analyzed the PHE scenario in India using the institutional and instructional reforms framework advanced by the Lancet Commission report (5) to understand the situation and advance the agenda of reforms for public health education in India.

Public health professionals have been defined broadly by Evashwick et al. (8) as *professionals of any discipline working in any aspect of the field of public health, including in government and non-government sectors, on a full-time or part-time basis*. Our working definition for public health professionals included only those who have a formal university level postgraduate qualification in public health. However, in the absence of any undergraduate course in public health in India, we included the undergraduate public health training as part of medical education (MBBS program).

The Lancet Commission on Education of Health Professionals identified three key components of the education system: institutional design (which specifies the structure and functions of the education system), instructional design (which focuses on processes), and educational outcomes (which deal with the desired results – outcomes, i.e., specific instructional objectives) (5, 19).We organized the search results according to the domains outlined in the Lancet Commission document (5).

In order to identify the current status of each individual domain under the framework, we conducted a systematic search that encompassed various published resources. These included visiting the central and state-level websites of the Ministries of Health and Family Welfare, web pages of donor agencies and multi-lateral agencies working in India, and website of the National Health Systems Resource Commission. We reviewed manuscripts for journals indexed in MedLine and snow-balled research articles through the reference lists of identified articles. We sought inputs from senior academicians and sought feedback on curriculum contents in their institutions. The information was entered into an Excel spreadsheet, organized according to the Lancet Commission framework.

The recommendations were framed to address the issues that emerged from the search. The evidence supporting the recommendation was cited wherever available.

## Current Status

If the education system is the supply side that fulfills the task of producing the health workforce, then the health system represents the demand side of the relationship. This relationship is complex in India, as the “public health system” is predominantly state supported. Over the past decade, the private sector (for-profit and not-for-profit) has gained a large share of the out-patient care as well as in-patient care (9). The limited workforce supply that emerges from the education sector thus fulfills the demand of an understaffed public health system as well as the demand of an ever expanding private health care. In short, here is an apparent disconnect between educational programs that produce public health professionals and the needs of the health systems.

The situation is further complicated by limited availability of job opportunities for public health graduates within the health system (10). This manifests even at the enrollment stage into public health programs. As an example, although the number of institutions that offer MPH programs in India has risen from 23 in 2011 (11) to 31 currently, the MPH courses reported a sub-optimal 75% enrollment in 2011 (11).

The traditional approach to public health education in India is through undergraduate and postgraduate training in medical colleges (6). The aim is to embed a problem solving and community outlook at both the undergraduate and the postgraduate level. Currently, there are 381 medical colleges in India, which produce about 49,668 medical graduates annually (12). Community medicine is a compulsory subject for all these medical undergraduates. The synergy between clinical medicine and the overarching common objective of providing comprehensive health care has been enshrined in the current undergraduate medical education, with an objective to train physicians capable of delivering primary health-care services (13).

The supply side of public health education was comprehensively examined for the first time in India (6) 2 years ago. In addition to the 49,000+ medical graduates produced annually, 218 institutions produce close to 740 post-graduates in community medicine/preventive and social medicine (12) and 45 institutions produce 151 diploma in public health (DPH)/diploma in community medicine annually (12). Little data exist about the employment of public health professionals across India, including graduates of formal training programs.

Table 1 outlines the number of institutions and their annual enrollment capacity. Unlike the West and in other developed countries, the contribution to the public health workforce by MPH degree holders is fairly limited. Currently, 31 institutes offer MPH programs in the country, producing 850 graduates annually. Over the last two decades, there has been a steady growth in the number of institutions offering MPH programs in India (6).

**Table 1:** **Enrollment capacity for select public health programs in India**.

Name of the program	Number of institutions	Enrollment capacity
MBBS (12)	381	49,668
MD (PSM/CM) (12)	218	740
DPH/DCM (12)	45	151
S health management/administration (6, 14)	51	2122
Hospital management/administration (6)	52	2500
Post graduate diploma in public health management (6, 15)	11	370
Occupational health (6, 16)	21	460
Epidemiology (6)	18	144
Distance education (17)	25 (69 courses)	Variable
Nutrition; public health nutrition (18)	190; 5	–
MPH	31	850

*Statistics depict the enrollment capacity, not actual enrolled or how many of the enrolled actually complete the program. It does not address the issue of student dropout/attrition*.

In India, the organization of health services extends from national to village level. At the national level, the Union Ministry of Health and Family Welfare plays the role of steward (6). At the state level, the organization is under the State Ministry of Health and Family Welfare. The medical and nursing programs are governed by their respective councils, but no single apex regulatory body governs the institutions offering programs that enroll non-medical graduates for public health programs. MPH programs across the country are governed by the universities with which these MPH schools are affiliated (11). The ownership of the medical schools is both public and private (not-for-profit) (6). The last two decades have witnessed a phenomenal rise in the private institutions offering undergraduate and postgraduate programs in medicine. However, governance structures are non-uniform across the institutions that offer public health programs in the absence of a public health council overseeing all these programs holistically.

An assessment of the tuition fee for MPH programs in India in 2011 revealed a large variation between public and private institutions offering the programs (11). The tuition fees for the entire program ranged from as low as 240 INR in a government supported institutes to as high 400,000 INR in a private institute (11, 21). A per-capita costing exercise in producing a public health graduate has not been undertaken for other disciplines, but will show a variation between public and private educational institutions (20).

Faculty is a key consideration in the quality of MPH instruction. In 1997, the Medical Council of India (MCI) mandatorily prescribed establishment of Medical Education Units (MEUs) to enable faculty members to avail modern education technology for teaching (22) and improve the quality of medical education by training the teachers (22). Additionally, through a network of 18 selected Regional Centers, MCI conducts Faculty Development Programs (22). However, this arrangement was under the auspices of the MCI and was restricted to the teaching faculty in medical colleges. Increasingly, MPH programs are now being offered outside the purview of medical colleges, and the faculty members of these MPH institutions do not have a structured program to avail modern education technology for teaching. A similar scenario exists in other institutions offering public health programs to non-medical graduates.

An overall faculty shortage exists across educational institutions in India, especially for specialized courses and higher education. The need is particularly severe in institutions offering public health programs. PhD programs in public health are limited, and no doctorate level programs similar to the DrPH program are available. This leads to an overall shortage in the relative availability of highly trained professionals to teach within public health schools or programs.

Although educational systems are deeply affected by both local and global contexts and many commonalities might be shared globally, there is local distinctiveness and richness (6). Such diversity provides opportunities for partnerships at an institutional level, or within the context of formal systems (6). This brings to the fore the important issue about the need to strengthen partnerships between different schools and also between schools and the health system. This is easier said than achieved in the educational setting in India (19). The governance structures of individual institutions are answerable only to their academic councils, which are housed within the corresponding University. In the absence of a formal structure and system of collaboration between Universities, there is little (if any) incentive for institutions to partner.

As a result, universities increasingly work in silos. Synergies are lacking that lead to innovations in the educational process and systems. Curricular reforms as well as changes in curricular delivery are sporadic and infrequently replicated. The ideal prospect for public health institutions is to link up with the health service delivery by adopting community blocks and establishing service areas linked to the public health education (5). This will facilitate a close collaboration with the health system and also provide an opportunity to the students for exposure to public health practice (5). Beyond the corridors of the medical institutions, institutions offering other professional public health courses would be expected to adhere to the university norms applicable to its affiliated institutions. This would be expected to be variable across different universities across the country. There is ample scope for strengthening partnerships between public health schools and between educational and other types of institutions (5, 19).

In addition to a shortage of faculty in general, there are neither set guidelines on the optimal student–faculty ratio for MPH programs, nor any guidelines stating the minimum specialties/departments within these institutions. Public health institutions would be optimally served in the presence of a strong multi-disciplinary team covering the core domains of public health. Currently, this is deficient, and there is relative opacity in the structuring of departments offering the public health courses. This challenge extends into training for other core infrastructure necessities, such as public health laboratory, public health museum, and the public health library.

With regard to student admissions, all institutions mandatorily follow reservation criteria as specified in the Indian constitution for the admission process. The MPH institutions offer seats on the basis of self-evolved university approved criteria. Admission criteria for other programs like the programs in health and hospital management and epidemiology exhibit great variability in admission criteria. These criteria are evolved by institutions with the permission and approval of the corresponding affiliated university in case of institutions affiliated to state universities. However, this may not be the circumstance in case of *deemed universities* who have their own criteria.

Curricular reforms are needed that reflect the modern educational thinking where competency-driven education must be at the center stage of the educational process (5). The end-product of the educational system should be a competent graduate who is able to perform effectively within the context of the public health system. Competency frameworks are currently missing, with educational institutions focusing either on knowledge–skills–attitudes or on problem solving. No formal platform exists to bring together the stakeholders to design the competency frameworks and the specific actual competencies that are expected of graduates.

Core competencies need to be identified for each curriculum domain, and each core competency should preferably have a skill based component added (6). Initial efforts have been undertaken under the auspices of the Indian Public Health Association with support from WHO–India, wherein an expert group developed a draft competency framework for public health professionals. Competency frameworks were proposed for various academic programs that included MD (Community Medicine/Preventive and Social Medicine), MBBS, Master of Public Health (MPH), Master of Health Management (MHM), and DPH. Other smaller initiatives toward competency-driven education have been undertaken by institutions singly and sometimes in a partnership (23). But although networks of individuals and of universities exist within India, their potential to bring about change has still not being utilized fully and effectively (19).

Availability of effective instruction modalities is a crucial component that can be either an opportunity or a challenge in competency acquisition. A strong presence of information-technology driven industry in India has resulted in modern teaching aids being near universal. Pedagogy in public health, however, is poorly documented, and little evidence documents a sustained impact of any particular technique on important public health outcomes at scale.

Currently, distance education programs in India have been largely limited to correspondence programs that involve the student receiving study material in the form of textbooks, course modules, or CDs that are then used for self-learning by the students (17), and the prospective students or the end users of the program are usually left out while designing such educational/training programs (17). However, there has been a positive change of late with leading distance-education courses adopting modern interactive interfaces (24–26).

The creation of job opportunities and designing career pathways for trained public health professionals is another area needing urgent attention (5). This is full-circle of the supply–demand relationships. Addressing this issue is extremely difficult as current service rules in the public health system across most states are not open to formally trained public health professionals unless they possess a medical or nursing background. The urgent need of an institutionalized public health service at central, state, and district levels, with clearly defined career pathways has been documented (27). Significant challenges pose entry into the public and private health sector, due to limited awareness of opportunities, lack of requisite skills for searching and applying for jobs and uncertain recruitment processes (27). One is not sure of the return on investments in terms of time, energies, and money invested. This is a crucial aspect to ensure sustainability. The absence of a well laid-out and structured career pathway was also a perceived barrier for entry into public health courses in the country.

## The Way Forward

The areas identified for action are wide in their scope and involve multiple stakeholders and ministries. While some (creating new institutions to create more human resources) are highly resource intensive, others involve a far greater discussion and joint planning (designing competency frameworks and creating career pathways for public health professionals). Hence no single agency can be currently tasked with addressing most (or all) of the activities.

The country was close to creating a National Commission for Human Resources in Health Bill, 2011 (28, 29), which would have provided a single governance and the architecture, as well as the forum for addressing these issues. However, the bill lapsed with the completion of the parliament term.

Against the context of an extensive physical infrastructure organized on the lines of the primary health-care approach, the country continues to face a shortage of manpower to respond to these public health challenges. Indian public health professionals function within the public as well as the private sector, their educational backgrounds are varied, and no single governing council spans across the governance spectrum for public health professionals. Both public and private educational institutions are engaged in educating public health professionals. The need for training an adequate number of professionals arises from within the health system, yet it has little formal say in the training of health professionals. The MCI can mandate the creation of a state-level body under the aegis of the State Medical Councils to facilitate a forum for dialogue between the health system and the training institutions (both in the public and in the private sector). This mechanism will need to be replicated across the multiple councils currently overseeing the education in both public and private sectors.

Public health graduates should be encouraged to work within health systems with well laid-out career pathways. Unlike the graduates from medicine or nursing within the public health sector who choose to work within the public health system, public health graduates find it difficult to be recruited and integrated into the mainstream public health system in the absence of a career pathway. To ensure that sufficient job opportunities are created, building a public health cadre in state health services would be a desirable and welcome step (6). The creation of a public health cadre is complicated as health is a state subject. As an initial step, the central government can take a lead pilot to demonstrate the creation of public health cadres in these select states, with a guide its adoption across other states in the country. Constant advocacy toward this issue has steadily sensitized health systems to increasingly opt for graduates from public health to find jobs in the public health system even in the absence of a public health cadre. However, this effort needs formal support in the form of a formal presence of a public health cadre.

The adoption of competency-driven education may not yet have a demonstrated impact on population health, yet its concept is highly encouraging. The current public health curriculum in India is not strongly competency-driven and is stuck in traditional teaching approaches. The teaching of public health is more complicated than that of a single clinical discipline given its strong inter-disciplinary leanings. Public health practice is best learned by doing, and problem-based learning is currently in vogue across several institutes. The competency-based education will be a logical extension of these efforts wherein the desired competencies for public health practice can be stated by the public health associations (like the Indian Public Health Association; Indian Association of Preventive and Social Medicine).

The adoption of trans-disciplinary and multi-school approaches in public health education curricula is not a recent idea and has been advocated by two Institute of Medicine reports in 2002 (30) and 2003 (31), and re-emphasized by Frenk et al. (5). The adoption and delivery of a competency-driven curriculum will not be an overnight transition. There is a distinctiveness in the philosophy of the teaching–learning efforts that hinges on the presence of a well-structured, rigorous, and regular effort to train the instructors. Instead of being looked upon as a cost-center, the development of an active and well-functioning teacher training program should be accorded top priority at the national and state level.

In the absence of a single accreditation council, there is diversity in the quality of the teaching experience, which will ultimately bear upon the quality of the graduate product. The current norms for medical and nursing colleges specified by their respective councils are prescriptive with a strong emphasis on infrastructure, while there is an absence of norms/criteria with regards to public health education. The professional councils will have to consciously undertake steps toward the creation of accreditation councils. These councils can have membership from the individual institutions offering the educational courses.

Resource generation, especially faculty development is more top-down in its approach, focuses more on short-term training workshops, with minimal efforts in documenting its impact. Given the plethora of private institutions engaged in producing public health professionals, the discussions on advancing the stewardship role of the ministry of health (or any other stakeholder) would meet limited success. The country finds itself in a situation where the public health system, which is government funded is in dire needs for higher numbers of public health professionals, but the educational institutions are either not fully geared-up to produce this high number (in the public sector), or has a limited incentive to initiate public health courses (in the private sector). Consequently, stop-gap arrangements through recruitment of other professionals in a contractual job agreement will be in vogue. In this complex scenario, the creation of a health-manpower planning unit within the ministry is an urgent necessity. This unit can be tasked with the compilation of current number of health professionals across the country, create a formal needs-assessment for the health systems (current as well as for the future), and suggest remedial measures for the country as a whole. The induction period for a public health professional to join the health system after completing the training is long and extends beyond the duration of their education. This will need consideration as a part of the planning process.

A well-trained public health student is the product of the right mixture of instructional and institutional factors. This in turn depends on the quality of the teaching–learning experience, which strongly depends on the instructors. Public health institutions will have to evolve strategies to also prevent the exodus of well-trained faculty, which may be due to low wages, poor support, or limited career prospects.

The technical know-how in information technology has percolated in the clinical care and clinical consultation through telemedicine, but is in a nascent stage when it comes to the teaching–learning experience. A structured development program in teaching–learning methods and pedagogy is needed. A nidus of faculty across disciplines who are trained in modern teaching–learning technology should be established in each institution. The presence of modern IT infrastructure has obvious application in offering distance-learning programs. The current needs of public health professionals cannot be addressed through purely traditional classroom-based chalk and talk methods of education and training (17). If the technological barrier has already been partly overcome, the strengthening of IT driven teaching–learning methods can be easily extended to offer focused, need-based courses of shorter duration. The under-tapped potential of distance education needs a serious re-look with introduction of quality-assured programs by reputed institutions at the earliest.

The emergence of a new century and our continued efforts as public health professionals to ensure equity in health may need us to re-visit the very core of the construct of professionalism in health care. While the very concept of *professionalism is* poorly understood, there has been much deliberation on this issue in recent times (32). Health professionals and those who train them are being challenged to work beyond their traditional comfort zones and often in teams and a new professionalism might be a mechanism for achieving improved outcomes by applying a “trans-disciplinary professionalism” (33).

## Conclusion

The future directions for public health education reforms in India must be strongly guided by these concerns. Current efforts within the country are sporadic, and urgent attention toward reforming public health education in its entirety is essential. Conventional stakeholders that should be engaged as a part of this transformative process include the students, the current workforce, the institutions (along with their academic staff), the health ministry, and the global agencies. However, the single most important stakeholder and beneficiary on successful initiation of the reforms process is the common public, which looks upon the public health system as a valuable and sometimes the only recourse in achieving an acceptable health status.

## Conflict of Interest Statement

The authors declare that the research was conducted in the absence of any commercial or financial relationships that could be construed as a potential conflict of interest.
